# Evaluating phone call follow-ups in Sub-Saharan Africa: A systematic review and meta-analysis

**DOI:** 10.1371/journal.pone.0334894

**Published:** 2025-10-27

**Authors:** Adesola Zadiat Musa, Folahanmi Tomiwa Akinsolu, Abideen Oluwarotimi Salako, Olunike Rebecca Abodunrin, Oluwabukola Mary Ola, Oliver Chukwujekwu Ezechi

**Affiliations:** 1 Clinical Sciences Department, Nigerian Institute Medical Research, Lagos, Nigeria; 2 Center for Reproduction and Population Health Studies, Nigerian Institute Medical Research, Lagos, Nigeria; 3 Department of Public Health, Faculty of Basic Medical and Health Sciences, Lead City University, Ibadan, Nigeria; 4 Global Health and Infectious Diseases, University of North Carolina at Chapel Hill, Chapel Hill, North Carolina, United States of America; 5 Department of Epidemiology, Centre for Global Health, School of Public Health, Nanjing Medical University, China; Medical Research Council / Uganda Virus Research Institute & London School of Hygiene and Tropical Medicine Uganda Research Unit, UGANDA

## Abstract

**Background:**

Healthcare systems in Sub-Saharan Africa (SSA) face significant challenges, including limited resources, understaffing, and geographical barriers, which hinder effective healthcare delivery. Phone call follow-ups have emerged as a promising strategy to improve participant retention, enhance data accuracy, and optimize health outcomes in resource-constrained settings. Despite their growing adoption, there is limited synthesized evidence of their effectiveness across various public health contexts in SSA.

**Methodology:**

This systematic review and meta-analysis included 32 studies published between 2000 and 2024, conducted in 11 SSA countries. Studies employing phone call follow-ups in community and facility-based health interventions were evaluated. Participant retention rates, reasons for loss to follow-up, and health outcomes were analyzed. Risk of bias and quality were assessed using validated tools tailored to study designs, including the Hoy et al. checklist for observational studies and the Joanna Briggs Institute (JBI) checklist for experimental studies. Statistical analysis employed a random-effects model to calculate pooled estimates and sensitivity analysis was conducted to assess the robustness of findings. Although the primary focus was on phone call follow-up interventions, a few included studies also utilized text messaging alongside phone calls.

**Results:**

The pooled retention rate across studies was 89% (95% CI: 85–91), with substantial variability among countries. Retention rates were highest in Kenya (96%) and Nigeria (87%). In contrast, countries like Cameroon reported a high participant loss rate of 42%. Frequent and consistent follow-up calls were associated with improved retention rates; studies that contacted participants 4–5 times reported retention rates as high as 98%. Barriers to follow-up included network issues, outdated contact information, and participant relocations. Risk of bias assessments showed that 81% of observational studies were rated as low risk. Additionally, 69% of experimental studies were assessed as high quality. Funnel plots assessing publication bias indicated some asymmetry in studies reporting lost rates, suggesting potential bias.

**Conclusion:**

Phone call follow-ups have enhanced participant retention and improved SSA health outcomes in regions with robust health infrastructure. However, variability in retention rates underscores the need for tailored strategies to address barriers like network challenges and participant mobility. Integrating innovative platforms like WhatsApp and leveraging consistent follow-up methods can enhance their scalability and impact. Policymakers should consider incorporating phone call follow-ups into routine care to optimize healthcare delivery in resource-constrained settings.

## Introduction

In Sub-Saharan Africa (SSA), healthcare systems face significant challenges, including inadequate infrastructure, understaffing, high patient loads, and geographical barriers to care [1–4]. Phone call follow-ups have emerged as an effective strategy to enhance healthcare delivery, particularly in resource-limited settings [5–7]. Phone call follow-ups involve structured interactions between healthcare providers and patients, either after facility-based interventions such as hospital discharge or as part of ongoing community-based programs [8–10]. These follow-ups aim to improve patient retention, address health concerns, and ensure adherence to treatment regimens [11–16]. While this review focuses primarily on phone call follow-ups, a few included studies also incorporated text messaging as a complementary method of participant engagement. This distinction is important given the differing technological and human resource implications (ref studies with phone calls).

Unlike face-to-face visits, which may be constrained by logistical, financial, and geographical challenges, phone call follow-ups offer a scalable solution to maintain continuity of care and enhance patient outcomes [4,17]. This approach has been used across various public health domains, including infectious diseases such as HIV/AIDS, tuberculosis, and malaria; non-communicable diseases such as diabetes and hypertension; maternal and child health; and behavioural health interventions. For example, phone call follow-ups have improved adherence to treatment plans, reduced hospital readmissions, and provided timely health education and support [18–20]. These benefits are particularly critical in SSA, where the fast-growing adoption of mobile phone technology offers a promising avenue for reaching underserved populations [18–32].

The importance of phone call follow-up interventions aligns with the WHO’s Global Strategy on Digital Health 2020–2025, which emphasizes the role of mobile health (mHealth) innovations in improving access to healthcare, enhancing efficiency within health systems, and empowering patients through better communication and information [3,4]. Studies in Kenya, Uganda, and South Africa have demonstrated the effectiveness of phone call follow-ups in diverse contexts, such as antenatal care, HIV management, and chronic disease monitoring [21,22,26,28,30–33]. For instance, phone call follow-ups among pregnant women in Kenya and South Africa have been associated with increased antenatal care attendance and skilled birth deliveries [31,34]. Similarly, in South Africa, phone call follow-ups have improved adherence to antiretroviral therapy and achieved higher viral suppression rates among individuals living with HIV/AIDS [30].

Despite these promising outcomes, there is limited synthesized evidence on the effectiveness of phone call follow-ups across different public health contexts in SSA. This gap is significant given the region’s unique healthcare challenges and the potential of mobile technology to address them [17,35,36]. This systematic review aims to understand their role in improving public health outcomes by examining the impact and implementation of phone call follow-ups. This review focused on assessing the efficacy of phone call follow-ups regardless of whether the initial intervention was facility-based or community-based. The findings could help identify best practices and inform the design of future interventions in resource-limited settings.

This review is particularly timely as it addresses an important question: How effective are phone call follow-ups in enhancing participant retention, ensuring data completeness, and improving health outcomes in SSA? By consolidating existing evidence, the study aims to bridge knowledge gaps and provide actionable insights for policymakers, researchers, and healthcare practitioners working to optimize healthcare delivery in the region.

## Methodology

### Ethical approval

Ethical approval was not required for this systematic review as the research was based on information retrieved from published studies.

### Study registration

This study followed the Preferred Reporting Items for Systematic Review and Meta-Analysis Review (PRISMA 2020) checklist [37]. A 27-item PRISMA checklist is available as in S1 File. This study protocol was registered in the International Prospective Register of Systematic Reviews (PROSPERO): CRD42024558559.

### Search strategy

The search strategy was designed to identify studies that reported on the use of phone calls follow-up strategies aimed at improving retention, adherence, or health service uptake in SSA by systematically searching literature databases (PubMed, Web of Science, and CINALH) and Google Scholar from January 2000 – June 2024. Detailed search strategies for each database are reported in S2 File. The bibliographies of relevant reviews and eligible studies were also examined for additional sources. Databases containing conference proceedings, congress’s annals, university theses, and experts were also consulted.

### Study selection

Following the initial search, all articles were loaded onto Rayyan Software (https://www.rayyan.ai/), where initial duplicate screening and removal were done [38]. After duplicate removal, the abstract and titles of retrieved articles were independently screened by two authors (FTA and ORA) based on pre-determined eligibility criteria. A second screening of full-text articles was also done independently by two authors (FTA and ORA). Disagreements during each screening stage were resolved through discussion and consensus by one of the authors (ZAM). The list of excluded studies and the reasons for their exclusion is presented in S3 File.

### Studies eligibility criteria

The eligibility criteria for the studies included in this systematic review on on the use of phone calls follow-up strategies aimed at improving retention, adherence, or health service uptake in SSA were carefully defined to ensure the selection of relevant and high-quality research. Table 1 shows the criteria for inclusion and exclusion parameters based on population, outcomes, and study design (PIOS framework).

**Table 1:** PICOS framework.

Component	Inclusion Criteria
Participants	• Participants of any age (children, adolescents, adults, elderly) involved in community-based studies. • Studies focusing on public health issues such as infectious, non-communicable, maternal and child health, or health behaviour interventions. • Studies conducted in SSA.
Intervention	• Phone call follow-ups conducted within community-based studies. • Follow-ups for participant retention, data collection, reminders, health education, or support. • Studies reporting on the frequency and duration of phone call follow-ups.
Comparator	• Not applicable
Outcomes	• Primary Outcomes: Participant retention rates; data completeness and accuracy. • Secondary Outcomes: Participant satisfaction.
Study Design	• Randomized Controlled Trials (RCTs). • Quasi-experimental studies. • Cohort studies. • Cross-sectional studies.

### Review question

**Primary Review Question:**

1. What is the effectiveness of phone call follow-ups in improving participant retention rates and health outcomes in SSA?

**Secondary Review Questions**:

1. What are the barriers and facilitators to implementing phone call follow-ups in healthcare interventions in SSA?
2. How do retention and loss rates vary across countries, as well as follow-up frequencies and intervention durations in SSA?

### PICOS framework

The PICOS Framework provides a structured approach for specifying the components of this systematic review, which evaluates the effectiveness of phone call follow-ups in studies conducted in SSA. (See Table 1).

### Study outcomes

We focused on four primary outcomes reported across the included studies:

1. Retention Rate: The proportion of participants who remained in the study and completed scheduled follow-up via phone calls.
2. Follow-up Rate: The percentage of participants successfully reached through phone calls for post-intervention or ongoing monitoring, regardless of full study completion.
3. Acceptability: The extent to which participants and/or implementers found the phone call follow-up strategy appropriate or satisfactory, as assessed through reported feedback or uptake indicators.
4. Feasibility: The practicality of implementing phone call follow-ups, including factors such as human resource demands, cost, technology use, and system integration, as reported in the original studies.

### Data extraction and analysis

#### Study selection.

We had a two-step screening process between two authors (FTA and ORA) working independently for all records. First, titles and abstracts were screened for eligible studies. Thereafter, for the eligible studies, full text was obtained for further review and final selection of eligible studies. Duplicates were removed using the Rayyan software [38]. We resolved disagreements regarding the inclusion of studies by discussing them or consulting a third review author (ZAM). We used the PRISMA flow chart to summarise the search and selection of studies for the review [39]. See the details of the Extraction in S4 File.

#### Data extraction.

The review team used a data extraction form adopted from the COCHRANE collaboration. We had a two-step extraction process between two authors (FTA and ORA) where each reviewer screened each record independently, after which the reviewers compared the outputs of each record, and where there was disagreement, an agreement was reached through discussions with an independent reviewer (ZAM). We extracted data on study design, location, sample size, retention rates, follow-up duration, and communication mode used (mode of follow-up). However, details about whether follow-up phone calls were conducted by health workers (human-led) or automated systems were not consistently reported across all studies.

### Quality assessment and risk of bias

The quality of included studies was assessed using validated tools tailored to each study design to ensure rigour and reproducibility in this systematic review. Two reviewers conducted The assessment process independently, and discrepancies were resolved through discussion or consultation with a third reviewer. The tools and methods used for quality assessment are described below. See the details of the Quality Assessment and Risk of Bias in S5 File.

1. For the observational studies, which include cross-sectional and cohort studies, the quality assessment by Hoy et al., 2012 was employed [40]. This tool evaluates studies across three domains: cohort selection, comparability of the groups, and outcomes assessment. Each study was assigned a score ranging from 0 to 9 stars, with higher scores indicating higher quality. Studies scoring 7 or more stars were categorized as high quality, 5–6 as moderate quality, and 4 or fewer as low quality [21,22,29,32,34,41–51].
2. The quality of experimental studies was evaluated using the Joanna Briggs Institute (JBI) Critical Appraisal Checklist for RCTs [52]. This tool examines key domains such as the adequacy of randomization, allocation concealment, blinding of participants and personnel, and the completeness of outcome data. Based on these criteria, studies were classified as low, moderate, or high risk of bias [23–28,30,31,33,53–59].

Inter-rater reliability (IRR) was calculated to ensure consistency and objectivity during the quality assessment [60]. The IRR score, measured using percentage agreement, was 89%, indicating a high level of agreement between the two reviewers. Discrepancies between reviewers were resolved through discussion, and if consensus could not be reached, a third reviewer was consulted. This process ensured the reliability and robustness of the quality assessment.

### Statistical analysis

We conducted a meta-analysis of studies that used phone calls for participant follow-up. Our assessment focused on the pooled retention rate and loss to follow-up by calculating the respective proportions. Statistical significance was defined at the 5% level, and summary statistics were presented as percentages with 95% confidence intervals (CIs) [61]. We employed a weighted approach to calculate the pooled prevalence, giving more weight to studies with larger sample sizes and more precise estimates. A random-effects model was used to account for the high heterogeneity anticipated across the studies, which was assessed using Cochrane’s Q-test and the inconsistency index (I2), with I2 values ≥50% indicating significant heterogeneity. We employed a random-effects model in the meta-analysis to account for the substantial heterogeneity (I2 = 98–99%) observed across studies. This approach is appropriate given the variability in populations and settings, allowing for the generalization of findings beyond the included studies and better reflecting the true variation across different contexts in SSA. We evaluated heterogeneity using Higgins’s I2, where an I2 greater than 50% indicated significant heterogeneity [62]. Forest plots were utilized to visualize the results [63]. We applied funnel plots to assess publication bias, and sensitivity analysis was performed using the leave-one-out method [64,65].

We further performed a subgroup analysis of retention rates by country, calculating the weighted proportion for each [61]. The duration of follow-up reported in each study was converted into weekly intervals to ensure consistent distribution, allowing us to analyze retention rates based on follow-up duration. The meta-analyses were executed using R version 4.3.3 (R Foundation for Statistical Computing), employing a random effects model (REM) for prevalence estimation [65,66].

### GRADE assessment of evidence

The Grading of Recommendations, Assessment, Development, and Evaluation (GRADE) framework was utilized to systematically assess the quality of evidence for key outcomes, including Retention Rate, Acceptability, Feasibility, and Follow-up Rates [37,38]. The methodology adhered to standardized GRADE protocols to evaluate evidence across the following five domains:

1. Risk of Bias: Each study was evaluated for methodological rigor, including the design (e.g., randomized controlled trials, cohort studies), participant recruitment, randomization, and blinding where applicable.
2. Inconsistency: Heterogeneity in study findings was assessed by comparing intervention effects across studies.
3. Indirectness: The applicability of the evidence to the target population and healthcare context was evaluated. This included assessing whether the study settings, populations, and interventions aligned with the intended use of phone call interventions in resource-limited settings.
4. Imprecision: Imprecision was assessed by reviewing confidence intervals, sample sizes, and event rates for key outcomes. Studies with small sample sizes or wide confidence intervals for effect estimates were downgraded due to insufficient precision in their findings.
5. Publication Bias: Potential publication bias was evaluated by examining reporting transparency, including the availability of protocols, consistency in reported outcomes, and the presence of funnel plot asymmetry where applicable.

### Assessment process

Two independent reviewers systematically assessed each included study against the GRADE domains, with disagreements resolved through discussion or consulting a third reviewer. Evidence for each outcome was assigned an initial certainty rating based on study design (e.g., high for randomized controlled trials and low for observational studies). The certainty was then downgraded or upgraded based on the domain assessments.

### GRADE rating and interpretation

Each outcome was assigned an overall certainty rating: high, moderate, low, or very low. The rating was determined by aggregating the evaluations across all five domains, following GRADE guidance:

- High certainty: The true effect is likely close to the estimated effect.
- Moderate certainty: The true effect is probably close to the estimate but may differ slightly.
- Low certainty: The true effect may be substantially different from the estimate.
- Very low certainty: There is significant uncertainty about the effect.

### Application to study outcomes

The GRADE framework was specifically applied to assess evidence for:

1. Retention Rate: The extent to which phone call interventions improved patient retention in care.
2. Acceptability: Participant satisfaction and perceived value of phone call interventions.
3. Feasibility: The practicality of implementing phone call interventions in diverse healthcare settings, considering resource availability.
4. Follow-up Rates: The effectiveness of phone calls in ensuring timely follow-up and continued engagement with healthcare services.

### Thematic analysis

Thematic analysis was utilized to extract key themes from the included studies [67]. Based on the findings, we identified several major themes, including satisfaction, preference, effectiveness, and reasons for participant attrition. These themes were then further organized into sub-themes based on their similarities.

## Results

### Selection of studies

**Fig 1:**
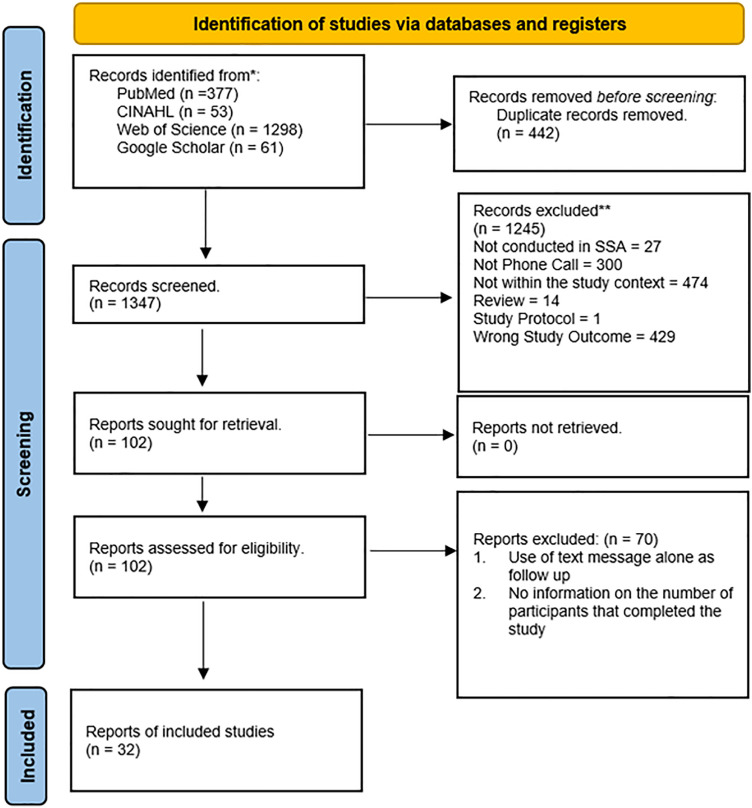
Prisma flow diagram.

Fig 1 illustrates the systematic study selection process. An initial search of three databases (PubMed, CINAHL, and Web of Science) and Google Scholar yielded 1789 articles from 2000 to 2024. After removing 650 duplicates, 1139 articles underwent title and abstract screening, excluding 1037 articles based on this study’s predefined exclusion criteria. The remaining 102 articles were subjected to full-text screening, finally leading to 32 articles [21–34,41–51,53–59] that met the predefined inclusion criteria.

### Characteristics of included studies

Table 2 shows synthesized data from 32 studies conducted across 11 African countries with 26,830 participants. Most studies were conducted in Kenya [22,26,28,34,55,58] and South Africa [21,30–32,43,56]. Four studies were conducted in Nigeria [24,51,53,54], three in Ghana [46,47,57], two in Cameroon [23,29], Tanzania [27,48], and one in Egypt [25], Ethiopia [59], Rwanda [50], Sudan [44], and Uganda [33]. Four studies involved multiple countries in Sub-Saharan Africa [42,49] and multiple countries: Zambia, Nigeria [41] and Namibia, Zambia, Nigeria and Uganda [45].

**Table 2:** Characteristics of included studies.

S/No	First Author (Year)	Country	Study Settings	Study Design	Sample Size	Mean age (years)	Follow-up Duration (Days)	Mode of follow-up	Health Program
1.	Abrahams et al., 2010 [30]	South-Africa	Facility	Experimental study	136	16	28	Phone calls	HIV Services (Post-exposure prophylaxis after rape)
2.	Asante et al., 2020 [57]	Ghana	Facility	Experimental study	60	55.07	90	Phone calls	Health Services (Type 2 Diabetes Management)
3.	Bigna et al., 2014 [23]	Cameroon	Facility	Experimental study	242	18	3	Text messages & Phone calls	HIV Care Programs
4.	Brown & Oluwatosin, 2017 [54]	Nigeria	Facility	Experimental study	305	14.6	390	Phone calls	Pediatric Health Services (Routine Immunization)
5.	Brunie et al., 2021 [41]	Nigeria, Zambia	Facility	Observational study	1542	33.3	365	Phone calls	Maternal and Child Health Services
6.	Chasimpha et al., 2022 [42]	Sub-Sahara Africa	Facility	Observational study	1635	31.7	1,095	Phone calls	Cancer Health Services
7.	Christie et al., 2020 [29]	Cameroon	Facility	Observational study	981	18	14	Phone calls	Injury Health Program
8.	Draaijer et al., 2020 [32]	South Africa	Facility	Observational study	400	28	332	Phone calls	HIV Care Services
9.	Du Preez et al., 2020 [43]	South Africa.	Facility	Observational study	395	11	7	Phone calls	Tuberculosis Referral Service
10.	Elbur et al., 2013 [44]	Sudan	Facility	Observational study	3656	13	–	Phone calls	Health Services (Wound Infections)
11.	Eldeib et al., 2019 [25]	Egypt	Facility	Experimental study	82	37.8	1,095	Phone calls	Cancer Health Services (Colorectal and Gastric cancer)
12.	Foerster et al., 2020 [45]	Namibia, Zambia, Nigeria, and Uganda	Facility	Observational study	1490	–	7	Phone calls	Cancer Health Services
13.	House et al., 2015 [22]	Kenya	Facility	Observational study	788	50	28	Phone calls	Pediatric Health Services
14.	Huchko et al., 2019 [55]	Kenya	Facility	Experimental study	930	5	270	Phone calls	Cancer Health Services (Cervical Cancer Screening)
15.	Ibraheem et al., 2021 [53]	Nigeria	Facility	Experimental study	140	37.4	90	Text messages & Phone calls	Pediatric Health Services
16.	Kababu et al., 2018 [58]	kenya	Facility	Experimental study	276	28.7	70	Phone calls	HIV Care Services (HIV Testing and Counselling)
17.	Kebaya et al., 2021 [28]	Kenya	Facility	Experimental study	75	28	28	Phone calls	Pediatric Health Services (HIV Care)
18.	Kukula et al., 2015 [46]	Ghana	Facility	Observational study	4124	25.9	–	Phone calls	Malaria Health Services
19.	Laar et al., 2019 [47]	Ghana	Facility	Observational study	489	29	56	Text messages & Phone calls	Maternal and Child Health Services
20.	Lyimo et al., 2024 [27]	Tanzania	Facility	Experimental study	175	32	1,295	Text messages & Phone calls	HIV Care Programs (Antenatal Care)
21.	MacKellar et al., 2018 [48]	Tanzania	Facility & community-based	Observational study	242	33.5	90	Phone calls	HIV Care Services (Linkage Sevices)
22.	McCormack et al., 2020 [49]	Sub-Saharan Africa	Facility	Observational study	305	37	1	Phone calls	Cancer Health Services
23.	Mugo et al., 2016 [26]	Kenya	Facility	Experimental study	1542	18	90	Text messages & Phone calls	HIV Care Programs
24.	Mutyambizi et al., 2022 [31]	South Africa	Facility	Experimental study	1635	23.5	2	Phone calls	Maternal and Child Health Services
25.	Mwaka et al., 2013 [34]	Kenya	Facility	Observational study	981	29.5	10	Phone calls	Health Services (Postoperative pain after surgery)
26.	Nkurunziza et al., 2022 [50]	Rwanda	Facility	Observational study	400	–	84	Phone calls	Maternal and Child Health Services
27.	Oladigbolu et al., 2020 [24]	Nigeria	Facility	Experimental study	395	30	56	Text messages & Phone calls	HIV Care Programs
28.	Parkes-Ratanshi et al., 2020 [33]	Uganda	Facility	Experimental study	3656	41.5	6,810	Phone calls	Maternal and Child Health Services (Syphilis Screening)
29.	Plazy et al., 2023 [56]	South-Africa	Facility	Experimental study	82	26	42	Phone calls	HIV Care Services (Linkage Services)
30.	Schwartz et al., 2015 [21]	South Africa	Facility	Observational study	1490	–	215	Text messages & Phone calls	HIV care programs
31.	Starr et al., 2020 [59]	Ethiopia	Facility	Experimental study	788	28	270	Phone calls	Health Services (Intra-operative Infection)
32.	Williams et al., 2021 [51]	Nigeria	Facility	Observational study	930	29.5		Phone calls	Pediatric Health Services (Surgery)

The included study designs were experimental [23–28,30,31,33,53–59] and observational studies [21,22,29,32,34,41–51]. Most of the studies followed up with participants via phone calls [22,25,28–34,41–46,48–51,54–59], while seven used a combination of text messages and phone calls [21,23,24,26,27,47,53]. The duration of follow-up varied widely, ranging from a day to 6,810 days [21–34,41–43,45,47–50,53–59]. Four studies conducted follow-ups for 90 days [26,48,53,57], and two studies each conducted follow-ups for the following number of days: 7 [43,45], 56 [24,47], 270 [55,59], and 1,095 [25,42]. The mean age of the study participants across the included studies ranged from 5 to 55.07 years [21–34,41–51,53–59]. The identity of the caller (human vs. automated) was not consistently reported in the included studies.

### Quality assessment and risk of bias of included studies

Of the 16 observational studies assessed [21,22,29,32,34,41–51], 81% (13/16) were rated as having a “low risk” of bias [21,22,32,34,43–51], demonstrating robust methodological quality in cohort selection, group comparability, and outcome assessment. The remaining 19% (3/16) were rated as “moderate risk” of bias [29,41,42], highlighting areas for improvement, particularly in comparability and outcome reporting. Overall, the predominance of low-risk ratings reinforces the reliability of findings from these studies.

Among the 16 experimental studies evaluated [23–28,30,31,33,53–59], 69% (11/16) were classified as “high quality,” reflecting adherence to key methodological criteria such as randomization, allocation concealment, and blinding [23–25,28,30,53–55,57–59]. The remaining 31% (5/16) were deemed “moderate quality,” pointing to areas for improvement, including outcome completeness and participant blinding [26,27,31,33,56]. Despite these limitations, most experimental studies exhibited strong internal validity, supporting confidence in their findings. (See details of the quality assessment and risk of bias in S5 File)

Table 3 provides an in-depth evaluation of the certainty of evidence for key study outcomes: Retention Rate, Acceptability, Feasibility, and Follow-up Rates.

**Table 3:** Summary GRADE assessment for study outcomes.

Outcome	Risk of Bias	Inconsistency	Indirectness	Imprecision	Publication Bias	Overall Certainty
**Retention Rate**	Low	Moderate	Minor	Moderate	Low	**Moderate**
**Acceptability**	Low	Low	Minor	Low	Low	**High**
**Feasibility**	Low	Low	Minor	Moderate	Low	**Moderate to High**
**Follow-up Rates**	Low	Moderate	Minor	Moderate	Low	**Moderate**

The certainty of evidence for the retention rate was rated moderate, primarily due to moderate inconsistency and imprecision in the findings. Although studies demonstrated consistent improvements in retention rates with interventions such as SMS reminders and phone calls, variability was observed across different settings. For instance, rural and resource-constrained areas showed lower retention rates than urban centers. Additionally, subgroup analyses lacked sufficient sample sizes to provide precise estimates, contributing to moderate imprecision. Despite these limitations, the evidence indicates that these interventions effectively enhance retention within healthcare systems.

For acceptability, the evidence was rated as high certainty, indicating strong and consistent findings across all included studies. Participants overwhelmingly found interventions like SMS reminders and phone calls convenient, accessible, and valuable in maintaining engagement with healthcare services. The absence of significant variability or methodological concerns further supports the reliability of these results. The high certainty of evidence underscores the acceptability of these interventions as a feasible strategy for promoting patient-centred care in diverse settings.

The certainty of evidence for feasibility was rated as moderate to high, reflecting robust findings but with some contextual limitations. The interventions were deemed feasible in urban and peri-urban areas with adequate infrastructure, including mobile phone penetration and healthcare system readiness. However, resource-constrained settings slightly reduced feasibility due to challenges such as poor network coverage, limited staff capacity, and lack of digital literacy. While the overall consistency of findings was strong, moderate imprecision was noted in some studies due to variability in resource availability and operational costs.

Finally, the evidence for follow-up rates was rated as moderate certainty. While interventions consistently improved follow-up rates, variability across healthcare systems and delivery mechanisms (e.g., SMS reminders versus in-person follow-ups) contributed to moderate inconsistency. Additionally, imprecision was noted due to wide confidence intervals and limited data from specific subgroups, such as populations with low mobile phone penetration. Nevertheless, the findings suggest that interventions can enhance follow-up rates, particularly in settings with reliable technological infrastructure.

### Quantitative analysis

#### Participants’ retention rate.

**Fig 2:**
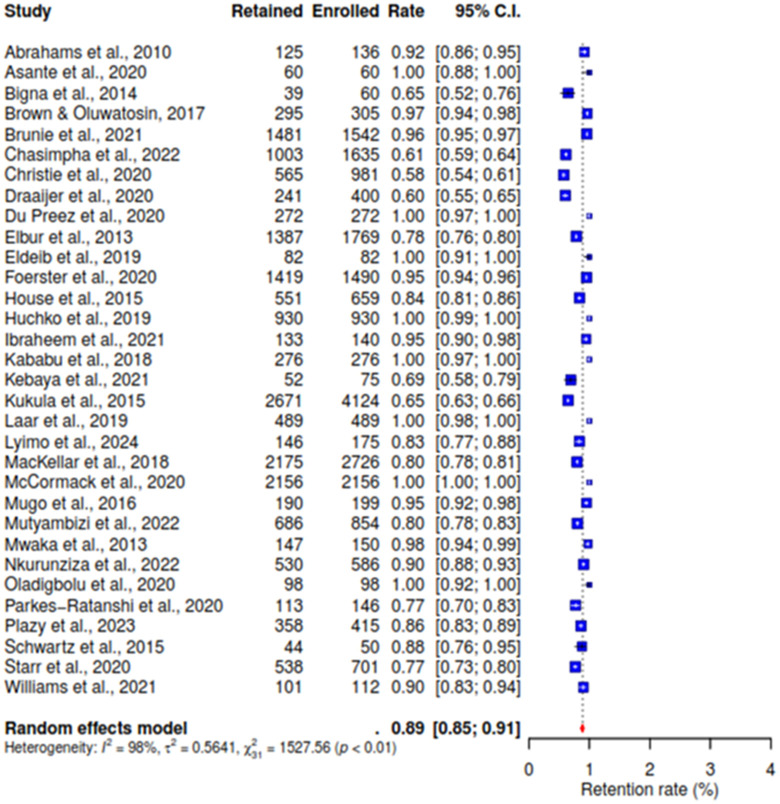
Forest plot of pooled participants’ retention rate.

This meta-analysis synthesizes data from 32 studies [21–34,41–51,53–59] to estimate the pooled retention rate among participants followed up via phone calls. The results show a pooled retention rate of 89% (95% CI: 85–91), indicating a high degree of participant retention when phone calls are used for follow-up. However, substantial heterogeneity was observed across studies I^^2^^ (98%), suggesting significant variability in retention rates between studies (See Fig 2).

**Fig 3:**
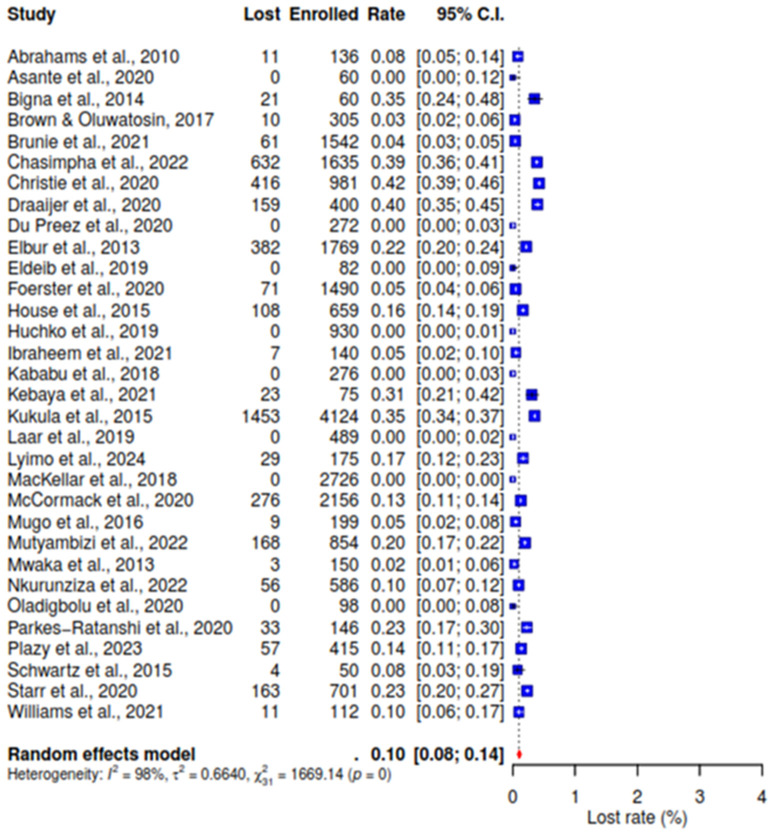
Forest plot of pooled participants’ loss to follow-up.

Conversely, the analysis reveals a low rate of lost participants from the 32 studies [21–34,41–51,53–59], at 10% (95% CI: 8–14), among the included studies that utilized phone calls for follow-up, indicating high participant retention with I^^2^^ value of 98% (See Fig 3).

#### Retention rate by countries.

**Fig 4:**
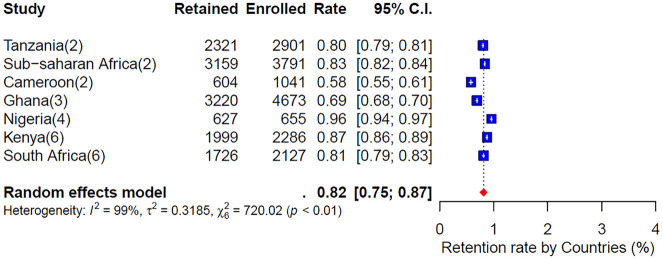
Weighted retention rate by countries.

Fig 4 illustrates the weighted retention and loss rates across countries, highlighting high participant retention rates in most countries in SSA. In Kenya, six studies reported a weighted retention rate of 96% and a loss rate of 4% [22,26,28,34,55,58]. Similarly, four studies from Nigeria showed a retention rate of 87% and a loss rate of 6% [24,51,53,54]. In Tanzania, two studies reported a retention rate of 80% and a loss rate of 1% [27,48]. Two studies from Cameroon reported a high loss rate of 42% and an average retention rate of 58% [23,29], while three studies from Ghana reported a loss rate of 31% and a retention rate of 69% [46,47,57].

#### Retention rate by duration of follow-up.

**Fig 5:**
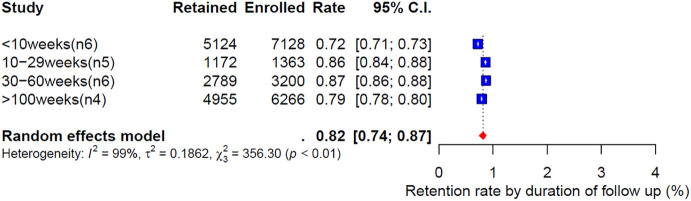
Weighted retention rate by the duration of phone call follow-up.

Fig 5 shows the weighted retention rates categorized by follow-up duration, revealing a consistently high retention rate across all duration categories. 72% for follow-up durations less than ten weeks [22–24,28–31,34,43–47,49,51,56], 86% for durations between 10 and 29 weeks [26,48,50,53,57,58], 87% for durations between 30 and 60 weeks [21,32,41,54,55,59], and 79% for follow-up periods exceeding 100 weeks [25,27,33,42].

#### Retention rate by frequency of phone calls.

**Fig 6:**
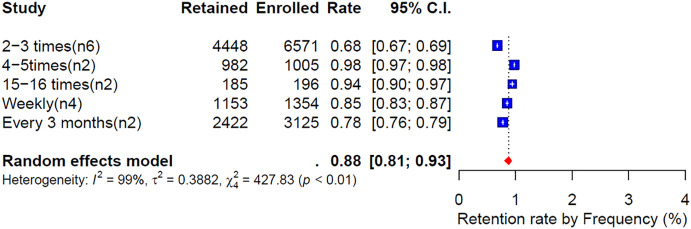
Weighted retention rate by frequency of phone call follow-up.

Fig 6 shows the weighted retention rates categorized by the frequency of phone calls, revealing a notable trend. Studies that called participants 4–5 times and 15–16 times reported high retention rates of 98% and 94%, respectively. In contrast, studies that called participants only 2–3 times had a significantly lower weighted retention rate of 68%.

#### Leave-one-out sensitivity analysis and publication bias.

The leave-one-out sensitivity analysis was performed to assess the robustness of the meta-analysis findings by recalculating the overall estimates after systematically removing each study, one at a time.

**Fig 7:**
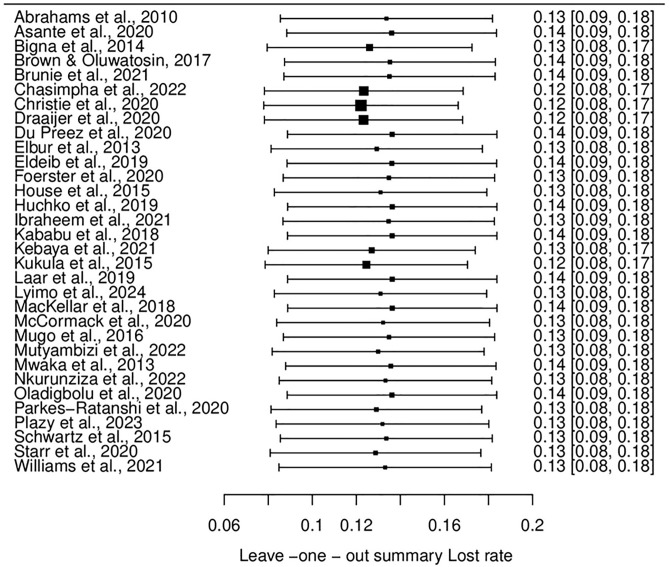
Leave-one-out analysis for loss rates.

**Fig 8:**
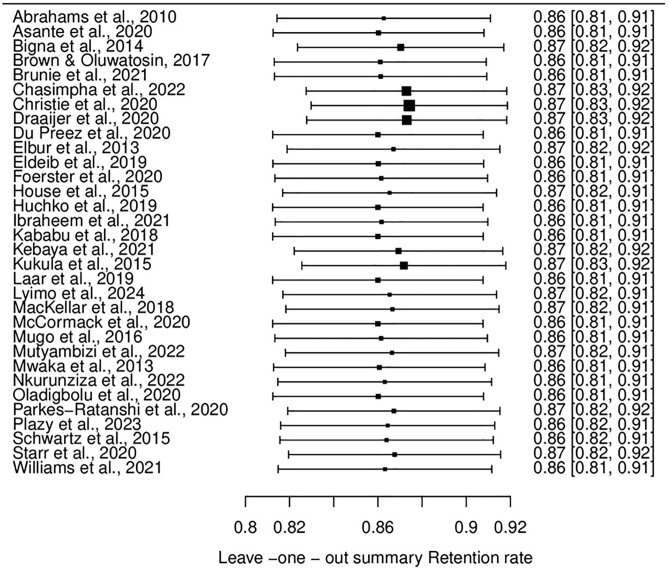
Leave-one-out analysis for retention rates.

The analysis for loss rates revealed that the pooled estimate remained relatively stable, ranging from 12% to 14% across iterations. This consistency indicates that no single study disproportionately impacted the overall findings. The pooled estimates’ stability underscores the reliability of the results for participant loss rates. (See Fig 7) Similarly, for retention rates, the leave-one-out analysis showed consistent pooled estimates, ranging from 86% to 87%, regardless of the study removed. The narrow range of variation in these estimates demonstrates robust findings, confirming that no individual study significantly influenced the overall retention rate. (See Fig 8)

**Fig 9:**
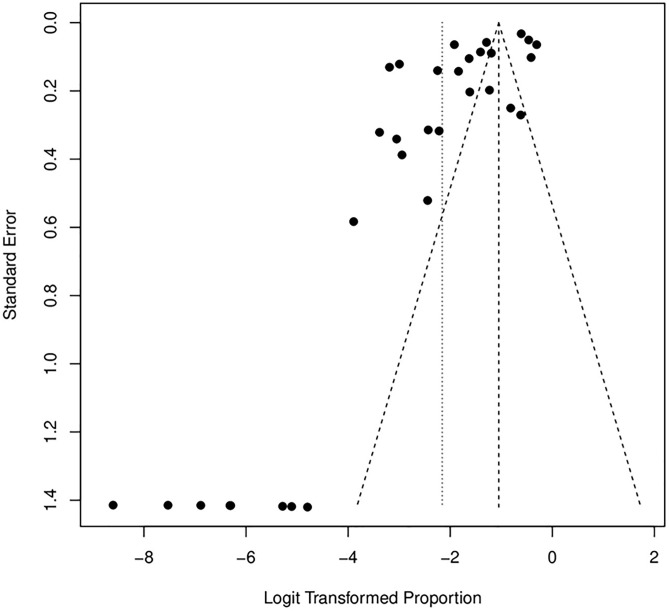
Funnel plot for loss rate.

**Fig 10:**
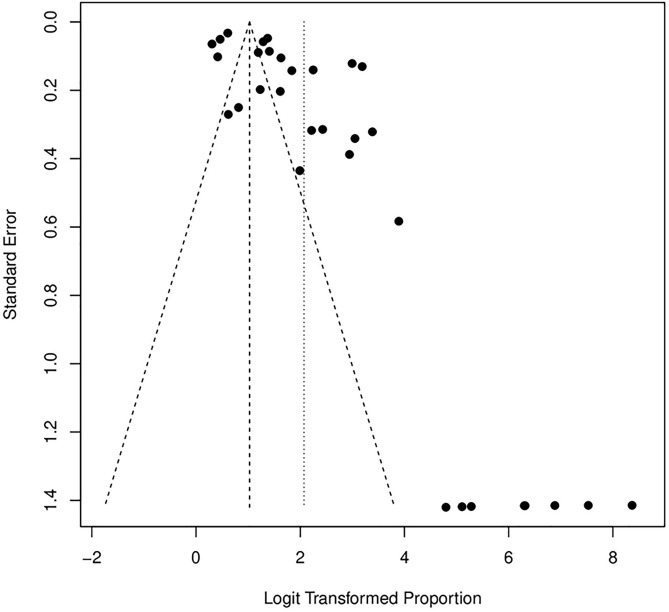
Funnel plot for retention rate.

The plot reveals noticeable asymmetry, which may indicate the presence of potential publication bias. Specifically, studies with smaller sample sizes and higher standard errors are unevenly distributed around the average effect. This pattern suggests that studies reporting higher loss rates may be underrepresented, potentially skewing the overall results. (See Fig 9) The retention rate funnel plot appears more symmetric, suggesting less evidence of publication bias in these studies. However, some dispersion is observed in the upper region, which may reflect variability in retention strategies across studies rather than systematic bias. This observation highlights the potential influence of diverse methodologies and intervention contexts on retention outcomes while indicating a more balanced representation of study findings. (See Fig 10)

### Thematic analysis of the included studies

This section presents the findings from the thematic analysis of 32 included studies [21–34,41–51,53–59] exploring various aspects of phone call follow-up methods in healthcare interventions. The analysis identified five key themes—**Satisfaction, Effectiveness, Feasibility, Adherence, and Cost-effectiveness**—and reasons for participant attrition. Table 4 summarises the themes, sub-themes, and reasons for participant loss.

**Table 4:** Shows the themes and sub-themes among the included studies.

Theme	Sub-theme
Satisfaction	–
Preference	–
Effectiveness	Feasibility
	Adherence
	Cost
Reason for loss of participants	Death
	Number unreachable
	Change of number
	Didn’t pick up the call
	Incorrect number
	Poor network coverage
	Non-functional phone
	Relocation
	Sick

#### Satisfaction.

Two studies found that participants expressed satisfaction with receiving phone calls [31,51,55]. In addition, three studies indicated a preference for phone call follow-ups among their participants [31,47,55].

#### Effectiveness.

Nineteen of the included studies [23–31,45,46,50,51,53,54,56–59] highlighted the effectiveness of phone call follow-ups. Two studies emphasized their utility in reminding participants [23,54], while one study noted its effectiveness in contacting participants [45]. Additionally, three studies reported increased uptake and improved vaccination rates attributed to this method [26,53,58]. One study observed reduced participant forgetfulness due to phone call follow-ups [24]. However, two studies found the method ineffective in their respective contexts [30,31].

#### Feasibility.

Three studies indicated that the phone call follow-up method is feasible and effective for data collection, providing patient follow-up care, and monitoring desired health outcomes [29,46,57].

#### Adherence.

Four studies found that most participants could adhere to their commitments due to phone call reminders [25,27,28,50].

#### Cost-effectiveness.

A study highlighted the affordability of the phone call follow-up method to effectively reach participants [59].

### Reason for loss of participants

An analysis of the 32 included studies identified several recurring themes related to participant attrition, with 16 studies reporting reasons for loss [22,23,26,27,29–32,42,45–47,49,51,54,56]. Two studies discussed participant deaths as a significant cause of participant loss [42,49]. Six studies reported that a notable number of participants became unreachable during the studies [22,26,29,31,46,47]. Four studies highlighted changes in participants’ contact numbers as a common reason for participant loss [22,27,30,32]. Some of the challenges highlighted were non-responsiveness to calls [23], encountered incorrect contact numbers [32], and faced challenges with non-functioning mobile phones [51]. One study mentioned participant sickness as a contributing factor [29], while two studies identified poor network coverage as a barrier to the loss of participants [51,56], and two studies reported participant relocation as a reason for participant loss [45,54].

## Discussion

This systematic review and meta-analysis evaluated the effectiveness of phone call follow-ups in improving participant retention and health outcomes across SSA. The findings highlight the potential of phone calls follow-up interventions to enhance healthcare delivery, as shown in 32 studies conducted between 2000 and 2024 in 11 African countries [21–34,41–51,53–59], particularly in countries like Kenya and South Africa. This geographic concentration underscores the importance of infrastructural capacity in implementing phone-based follow-ups effectively [3,4].

### Retention rates and communication strategies

Retention rates varied significantly across countries and follow-up durations, reflecting the diverse challenges faced in SSA. Frequent contact was consistently associated with higher retention rates, reinforcing the critical role of consistent communication in fostering participant trust and engagement [68,69]. Conversely, infrequent follow-ups correlated with lower retention rates, highlighting the dual importance of both the frequency and the quality of communication. By “quality of communication,” we refer to the interpersonal aspects of the phone interaction, such as empathy, clarity, cultural sensitivity, and responsiveness, which are essential for building rapport and addressing participants’ concerns during follow-up. While comparisons to global evidence demonstrate similar trends [23,30,57,70,71], the findings in SSA underscore the unique importance of sustained contact in settings where access to healthcare is often limited [30,54,71,72].

### Barriers to implementation

This review identified key barriers to implementing phone call follow-ups in SSA, including network connectivity issues, outdated or incorrect contact information, and participant relocations. While these challenges are not exclusive to resource-constrained settings, they are often more pronounced due to infrastructural and systemic limitations, such as limited access to electronic medical records, frequent changes in phone numbers due to SIM instability, and inconsistent updating of patient contact details [1,29,72]. Additionally, integrating alternative communication platforms such as WhatsApp may offer a more accessible and cost-effective means of improving follow-up success rates. Unlike traditional phone calls, WhatsApp supports asynchronous communication, enabling participants to respond at their convenience, which is particularly useful in settings with intermittent connectivity or work-related constraints. It also allows for multimedia messaging, such as text reminders, audio prompts, and educational images or videos, which may enhance engagement and understanding. WhatsApp’s widespread adoption across SSA among both healthcare providers and patients adds to its feasibility and acceptability [70,73–76]. Future studies should assess the feasibility of blended approaches, combining basic mobile phone calls with app-based platforms, based on local infrastructure and population access.

### Adherence and satisfaction

Phone call follow-ups were found to improve participant adherence and satisfaction significantly. Personalized communication fostered a sense of connection and trust, motivating participants to adhere to interventions [73,77]. This effect was particularly evident in chronic disease management and post-operative care, where sustained engagement is critical for positive outcomes [72,78]. The SSA-specific evidence aligns with global findings, reinforcing the value of individualized follow-ups in maintaining long-term adherence and satisfaction [74].

### Feasibility and cost-effectiveness

The feasibility and cost-effectiveness of phone call interventions were strongly supported in this review. Phone-based follow-ups demonstrated significant cost savings compared to in-person visits and were feasible even in resource-limited settings [29,46,54,79]. However, the variability in feasibility and cost-effectiveness across SSA highlights the need for tailored strategies. Interventions must be adapted to specific populations and healthcare systems, considering factors like infrastructure, resource allocation, and the target population [4,74,76,78,80].

### Implications for practice and policy

The findings underscore the potential of phone call follow-ups to bridge healthcare gaps in SSA. Policymakers and healthcare providers should prioritize integrating phone-based interventions into routine care, particularly chronic disease management and maternal health programs [3,19,23,28,30,32,35,69,77]. The scalability of such approaches depends on addressing barriers like network connectivity and leveraging innovative technologies. Future research should optimize follow-up strategies, harmonize regional practices, and explore integrating digital platforms like WhatsApp to enhance scalability and sustainability [3,4].

This study offers significant insights into the effectiveness of phone call follow-ups in improving participant retention and health outcomes across SSA. A key strength lies in its comprehensive scope, synthesizing evidence from 32 studies across 11 SSA countries. A rigorous methodology, including validated tools for quality assessment, enhances the credibility of the findings. The study highlights actionable strategies to address barriers to retention, offering practical implications for policymakers and healthcare providers. These include verifying contact information at enrolment, using structured follow-up schedules, offering modest incentives (e.g., airtime), ensuring culturally appropriate communication, and integrating platforms like WhatsApp. Assigning dedicated follow-up staff also proved effective. These approaches can guide implementers in optimizing follow-up in resource-limited settings.

However, several limitations should be noted. The geographic focus on Kenya and South Africa limits generalizability to other SSA countries with less developed mHealth infrastructure. Variability in study designs and potential publication bias may affect the comparability and reliability of pooled results. Furthermore, limited data on long-term health outcomes and the underrepresentation of innovative platforms like WhatsApp constrain the study’s ability to evaluate the sustainability and scalability of phone call follow-ups. Future research should address these gaps to strengthen the evidence base.

## Conclusion

This systematic review and meta-analysis demonstrated the potential of phone call follow-ups to improve SSA participant retention and health outcomes. High retention rates in Kenya and Nigeria highlight the effectiveness of robust follow-up strategies, though variability across countries underscores the need for tailored approaches to address challenges like network issues and outdated contact information. While the findings align with global evidence on mHealth interventions, the geographic concentration in Kenya and South Africa limits generalizability. Frequent and consistent communication remains critical for engagement, and integrating innovative platforms like WhatsApp could enhance scalability and effectiveness in resource-constrained settings.

## Supporting information

S1File PRISMA checklist. (PDF)

S2 File Search strategy. (PDF)

S3 File Excluded studies. (XLSX)

S4 File Extraction sheet. (XLSX)

S5 File Quality assessment and risk of bias. (XLSX)
