# Supplementary material for: Evaluating phone call follow-ups in Sub-Saharan Africa: A systematic review and meta-analysis
Source: PLoS One. 2025 Oct 27;20(10):e0334894. doi: 10.1371/journal.pone.0334894 (PMC12558458; doi:10.1371/journal.pone.0334894)
Supplement: S2 File — (PDF) [file pone.0334894.s002.pdf]

Can they use mobile phone call or text message as Follow up and how effectively can it be?

Pubmed- 377

Search	Search term	Search strategy	Results
#1	Phone call	"Phone calls"[Title/Abstract] OR "Phone call"[Title/Abstract] OR Telephonic[Title/Abstract] OR Telephone[Title/Abstract] OR "Mobile phone"[Title/Abstract] OR "Telephone calls"[Title/Abstract] OR "Text messaging"[Title/Abstract] OR "Text message"[Title/Abstract] OR text[Title/Abstract] OR SMS[Title/Abstract]	225,455
#2	Follow-up	follow-up[Title/Abstract] OR "Follow up"[Title/Abstract] OR reminders[Title/Abstract] OR reminder[Title/Abstract]	1,288,222
#3	#1 AND #2	("Phone calls"[Title/Abstract] OR "Phone call"[Title/Abstract] OR Telephonic[Title/Abstract] OR Telephone[Title/Abstract] OR "Mobile phone"[Title/Abstract] OR "Telephone calls"[Title/Abstract] OR "Text messaging"[Title/Abstract] OR "Text message"[Title/Abstract] OR text[Title/Abstract] OR SMS[Title/Abstract]) AND (follow-up[Title/Abstract] OR "Follow up"[Title/Abstract] OR reminders[Title/Abstract] OR reminder[Title/Abstract])	28,257
#4	Sub-saharan	Algeria[Title/Abstract] OR Angola[Title/Abstract] OR Benin[Title/Abstract] OR Botswana[Title/Abstract] OR Burkina Faso[Title/Abstract] OR Burundi[Title/Abstract] OR Cabo Verde[Title/Abstract] OR Cameroon[Title/Abstract] OR Central African Republic[Title/Abstract] OR Chad[Title/Abstract] OR Comoros[Title/Abstract] OR Democratic Republic of the Congo[Title/Abstract] OR Djibouti[Title/Abstract] OR Egypt[Title/Abstract] OR Equatorial Guinea[Title/Abstract] OR Eritrea[Title/Abstract] OR Eswatini[Title/Abstract] OR Ethiopia[Title/Abstract] OR Gabon[Title/Abstract] OR Gambia[Title/Abstract] OR Ghana[Title/Abstract] OR Guinea[Title/Abstract] OR Guinea-Bissau[Title/Abstract] OR Ivory Coast (Côte d'Ivoire[Title/Abstract]) OR Kenya[Title/Abstract] OR Lesotho[Title/Abstract] OR Liberia[Title/Abstract] OR Libya[Title/Abstract] OR Madagascar[Title/Abstract] OR Malawi[Title/Abstract] OR Mali[Title/Abstract] OR Mauritania[Title/Abstract] OR Mauritius[Title/Abstract] OR Morocco[Title/Abstract] OR Mozambique[Title/Abstract] OR Namibia[Title/Abstract] OR Niger[Title/Abstract] OR Nigeria[Title/Abstract] OR Republic of the Congo[Title/Abstract] OR Rwanda[Title/Abstract] OR São Tomé[Title/Abstract] AND Príncipe[Title/Abstract] OR Senegal[Title/Abstract] OR Seychelles[Title/Abstract] OR Sierra Leone[Title/Abstract] OR Somalia[Title/Abstract] OR South Africa[Title/Abstract] OR South Sudan[Title/Abstract] OR Sudan[Title/Abstract] OR Tanzania[Title/Abstract] OR Togo[Title/Abstract] OR Tunisia[Title/Abstract] OR Uganda[Title/Abstract] OR Zambia[Title/Abstract] OR Zimbabwe[Title/Abstract]	126,141
#5	#3 AND #4	(("Phone calls"[Title/Abstract] OR "Phone call"[Title/Abstract] OR Telephonic[Title/Abstract] OR Telephone[Title/Abstract] OR "Mobile phone"[Title/Abstract] OR "Telephone calls"[Title/Abstract] OR "Text messaging"[Title/Abstract] OR "Text message"[Title/Abstract] OR text[Title/Abstract] OR SMS[Title/Abstract]) AND (follow-up[Title/Abstract] OR "Follow up"[Title/Abstract] OR reminders[Title/Abstract] OR	377

		reminder[Title/Abstract])) AND (Algeria[Title/Abstract] OR Angola[Title/Abstract] OR Benin[Title/Abstract] OR Botswana[Title/Abstract] OR Burkina Faso[Title/Abstract] OR Burundi[Title/Abstract] OR Cabo Verde[Title/Abstract] OR Cameroon[Title/Abstract] OR Central African Republic[Title/Abstract] OR Chad[Title/Abstract] OR Comoros[Title/Abstract] OR Democratic Republic of the Congo[Title/Abstract] OR Djibouti[Title/Abstract] OR Egypt[Title/Abstract] OR Equatorial Guinea[Title/Abstract] OR Eritrea[Title/Abstract] OR Eswatini[Title/Abstract] OR Ethiopia[Title/Abstract] OR Gabon[Title/Abstract] OR Gambia[Title/Abstract] OR Ghana[Title/Abstract] OR Guinea[Title/Abstract] OR Guinea-Bissau[Title/Abstract] OR Ivory Coast (Côte d'Ivoire[Title/Abstract]) OR Kenya[Title/Abstract] OR Lesotho[Title/Abstract] OR Liberia[Title/Abstract] OR Libya[Title/Abstract] OR Madagascar[Title/Abstract] OR Malawi[Title/Abstract] OR Mali[Title/Abstract] OR Mauritania[Title/Abstract] OR Mauritius[Title/Abstract] OR Morocco[Title/Abstract] OR Mozambique[Title/Abstract] OR Namibia[Title/Abstract] OR Niger[Title/Abstract] OR Nigeria[Title/Abstract] OR Republic of the Congo[Title/Abstract] OR Rwanda[Title/Abstract] OR São Tomé[Title/Abstract] AND Príncipe[Title/Abstract] OR Senegal[Title/Abstract] OR Seychelles[Title/Abstract] OR Sierra Leone[Title/Abstract] OR Somalia[Title/Abstract] OR South Africa[Title/Abstract] OR South Sudan[Title/Abstract] OR Sudan[Title/Abstract] OR Tanzania[Title/Abstract] OR Togo[Title/Abstract] OR Tunisia[Title/Abstract] OR Uganda[Title/Abstract] OR Zambia[Title/Abstract] OR Zimbabwe[Title/Abstract])	

Cinahl- 53

Google scholar- 61

Web of science=1298

SN	Key words	Search strategy	
1	Phone call	**((((((((((((TS=(Phone call)) OR TS=(Telephonic)) OR TS=(Telephone)) OR TS=(Telehealth)) OR TS=(Mobile phone)) OR TS=(Telecommunication)) OR TS=(mhealth)) OR TS=(text)) OR TS=(SMS)) OR TS=(Text messaging)) OR TS=(Text message)) OR TS=(Phone calls)) OR TS=(telephone calls)** and **Preprint Citation Index** (Exclude – Database)	**9793317**
2	Follow-up	**(((TS=(reminder)) OR TS=(reminders)) OR TS=(Follow up)) OR TS=(follow-up)** and **Preprint Citation Index** (Exclude – Database)	**3062849**
3	sub-Sahara Africa	#2 AND #1 and Preprint Citation Index (Exclude – Database) and SOUTH AFRICA or KENYA or UGANDA or NIGERIA or ETHIOPIA or EGYPT or TANZANIA or GHANA or SIERRA LEONE or COTE IVOIRE or DEM REP CONGO or BOTSWANA or TUNISIA or RWANDA or MOZAMBIQUE or ZIMBABWE or ZAMBIA or BURKINA FASO or CAMEROON or LESOTHO or BENIN or LIBYA or TOGO or LIBERIA or REP CONGO or SWAZILAND (Countries/Regions) and 2024 or 2023 or 2022 or 2021 or 2020 or 2019 or 2018 or 2016 or 2017 or 2015 or 2014 or 2013 or 2012 or 2011 or 2010 or 2009 or 2008 or 2007 or	1298

		2006 or 2005 or 2004 or 2003 or 2002 or 2001 or 2000 (Publication Years) and Review Article (Exclude – Document Types)	
