# Supplementary material for: Evaluating phone call follow-ups in Sub-Saharan Africa: A systematic review and meta-analysis
Source: PLoS One. 2025 Oct 27;20(10):e0334894. doi: 10.1371/journal.pone.0334894 (PMC12558458; doi:10.1371/journal.pone.0334894)
Supplement: Attachment — Submitted filename: Response to Reviewer 2.pdf [file pone.0334894.s007.pdf]

Response to Reviewer 2

Reviewer #2: Overall this is an interesting article and a lot of effort was put into the analysis. It is however somewhat lacking in the discussion area and claims to share more insight than it does.

1. In the abstract and the text you write that:
“Retention rates were highest in Kenya (96%) and Nigeria (87%), while countries like Cameroon reported a loss rate of 42%.”
and
“Risk of bias assessments revealed that 81% of observational studies were low risk, while 69% of experimental studies were rated high quality.”
I would suggest both sentences be made into two sentences for clarity – otherwise the reader tries to compare retention and loss, or risk and high quality.

Response:
Thank you for your insightful comment. We agree that separating the statements will improve clarity and prevent unintended comparisons. We have revised the relevant sentences in the abstract as follows:
“Retention rates were highest in Kenya (96%) and Nigeria (87%). In contrast, countries like Cameroon reported a high participant loss rate of 42%.”
“Risk of bias assessments showed that 81% of observational studies were rated as low risk. Additionally, 69% of experimental studies were assessed as high quality.”

2. The Search strategy and Studies Eligibility Criteria both mention West Africa – is it not SSA?
Response:
Thank you for pointing this out. We have reviewed and corrected all instances to reflect Sub-Saharan Africa (SSA) in both the Search Strategy and Studies Eligibility Criteria sections to ensure consistency with the scope of the review.

3. You mention in the methods and results sections that text messages were sometimes included. It should be made clear in the abstract and introduction of the paper that text messages were included in some studies - text and phone calls use different software and incur different human/tech costs.
Response:
Thank you for this valuable suggestion. We agree that phone calls and text messages involve different implementation considerations and costs. While our review primarily focused on studies using **phone call follow-up** interventions, a small number of the included studies incorporated **text messaging** alongside phone calls.
To improve clarity and transparency, we have now updated the **Introduction** to briefly note that some studies included text messaging as part of a broader phone-based intervention. We have also added a line in the **Abstract** to acknowledge this, while maintaining the focus on phone call follow-up as the primary intervention of interest.

4. Please also make clear if the phone calls were all via a health worker (human), an automated system or a combination of both.
Response:
Thank you for this important observation. We have clarified in both the **Methods** and **Results** sections that the **mode of phone call delivery (i.e., human vs. automated)** was not consistently reported across the included studies. As such, we did not provide a separate analysis or summary on this aspect.

5. Please define: Retention Rate, Acceptability, Feasibility, Follow-up Rates early in the paper
Response:
Thank you for this important suggestion. We have now provided clear operational definitions of the key outcomes — *retention rate, acceptability, feasibility, and follow-up rate* — in the **Methods section under a new subsection titled "Study Outcomes."** This placement ensures that readers are aware of how these outcomes were conceptualized and measured before encountering the results.

6. PICOS misspelled lines 114, 125, 129
Response:
Thank you for pointing this out. We have corrected the spelling of **PICOS** in all indicated lines. Regarding the "Comparator" component, we have updated the PICOS framework table and narrative to reflect that **no comparator was applicable** in our review, and this is now clearly stated as *"Comparator: Not applicable."*

7. Table 2: Can you please categorize the papers further? There is a column for 'Study settings'. We need a column for type of health program e.g. chronic disease management, maternal health programs
Response
Thank you. We have added a column for health programs.

8. You reference 'mHealth infrastructure' in lines 419 and 466. This is this something different than simply network connectivity'? Clarity needed.
Response:
Thank you for your helpful comment. We agree that the term "mHealth infrastructure" may have introduced ambiguity, especially since the primary focus of our review is on phone call follow-up interventions. To address this, we have revised the text to more accurately reflect our intended meaning.

9. Lines 425-426: you write 'Conversely, infrequent follow-ups correlated with lower retention rates, emphasizing the dual importance of both frequency and quality of communication?' There is nothing mentioned on quality – please clarify.
Response

Thank you for your thoughtful comment. We acknowledge that the term *“quality of communication”* could be clearer in the context of our study. In this review, we used it to refer to the interpersonal effectiveness of the follow-up interaction, such as the clarity, empathy, and responsiveness of the health worker during the phone call, rather than technical aspects like call quality or signal strength.
To address this, we have revised the text to clarify that “quality of communication” refers to how well the phone interaction fosters trust, understanding, and patient engagement, which is particularly important in contexts where patients may have concerns or questions that require human reassurance. We have made this distinction explicit in the relevant paragraph to improve clarity and alignment with the study’s focus on human-led phone call follow-ups.

10. Line 431-433: you write “This review identified key barriers to implementing phone call follow-ups, including network connectivity issues, outdated or incorrect contact information, and participant relocations. These challenges, specific to resource-constrained environments, highlight the need for robust participant management strategies.” Are outdated and or incorrect contact information and participant relocations actually specific to resource-constrained environments? And can you give example(s) of a robust participant management strategy?
Response:
Thank you for this insightful comment. We agree that outdated or incorrect contact information and participant relocations are not unique to resource-constrained settings and may occur in various contexts. However, in resource-limited environments, these challenges are often compounded by systemic factors such as inconsistent record-keeping practices, lack of electronic medical records, and limited mobile phone ownership or SIM card stability (e.g., frequent number changes). We have revised the sentence to reflect this nuance more accurately.

11. Line 435: you write “Additionally, integrating alternative communication platforms like WhatsApp could provide more accessible, cost effective options to improve follow-up success rates [70, 73-76].” This seems to be your big takeaway, so more needs to be added on why this is beneficial and how implementers could take this advice into account.
Response:
Thank you for this helpful observation. We agree that the mention of alternative communication platforms like WhatsApp warrants further elaboration, especially given its widespread use and growing utility in healthcare delivery across Sub-Saharan Africa.
We have revised the text to better explain why integrating platforms such as WhatsApp may be beneficial. Specifically, we note that WhatsApp allows for asynchronous communication, reduced costs compared to voice calls, and the ability to share multimedia content (e.g., images, reminders, or educational material), which can enhance patient engagement. Furthermore, WhatsApp is already familiar to both healthcare providers and patients in many African settings, making it a feasible and acceptable tool for follow-up.

12. Line 463: You write “The study highlights actionable strategies to address barriers to retention, offering practical implications for policymakers and healthcare providers.” I do not

think the discussion shares enough actionable strategies. More examples and context from the reviewed studies would strengthen this article.

Response:

Thank you for the helpful suggestion. We have revised the discussion to include concrete examples of actionable strategies such as verifying contacts, scheduled follow-ups, small incentives, local language use, WhatsApp integration, and dedicated follow-up staff, to enhance practical relevance for implementers.
