# Supplementary material for: Evaluating phone call follow-ups in Sub-Saharan Africa: A systematic review and meta-analysis
Source: PLoS One. 2025 Oct 27;20(10):e0334894. doi: 10.1371/journal.pone.0334894 (PMC12558458; doi:10.1371/journal.pone.0334894)
Supplement: Peer review history

# Peer review: Decision Letter 0

**DOI:** 10.1371/journal.pone.0334894.r002

**Authors:** Francis Xavier Kasujja

21 Aug 2025

Dear Dr. Akinsolu,

Thank you for submitting your manuscript to PLOS ONE. After careful consideration, we feel that it has merit but does not fully meet PLOS ONE’s publication criteria as it currently stands. Therefore, we invite you to submit a revised version of the manuscript that addresses the points raised during the review process.

Please submit your revised manuscript by Oct 05 2025 11:59PM. If you will need more time than this to complete your revisions, please reply to this message or contact the journal office at plosone@plos.org . When you're ready to submit your revision, log on to https://www.editorialmanager.com/pone/ and select the 'Submissions Needing Revision' folder to locate your manuscript file.

- A rebuttal letter that responds to each point raised by the academic editor and reviewer(s). You should upload this letter as a separate file labeled 'Response to Reviewers'.
- A marked-up copy of your manuscript that highlights changes made to the original version. You should upload this as a separate file labeled 'Revised Manuscript with Track Changes'.
- An unmarked version of your revised paper without tracked changes. You should upload this as a separate file labeled 'Manuscript'.

If applicable, we recommend that you deposit your laboratory protocols in protocols.io to enhance the reproducibility of your results. Protocols.io assigns your protocol its own identifier (DOI) so that it can be cited independently in the future. For instructions see: https://journals.plos.org/plosone/s/submission-guidelines#loc-laboratory-protocols . Additionally, PLOS ONE offers an option for publishing peer-reviewed Lab Protocol articles, which describe protocols hosted on protocols.io. Read more information on sharing protocols at https://plos.org/protocols?utm_medium=editorial-email&utm_source=authorletters&utm_campaign=protocols .

We look forward to receiving your revised manuscript.

Kind regards,

Francis Xavier Kasujja

Academic Editor

PLOS ONE

Journal Requirements:

When submitting your revision, we need you to address these additional requirements.

1. Please ensure that your manuscript meets PLOS ONE's style requirements, including those for file naming. The PLOS ONE style templates can be found at 

https://journals.plos.org/plosone/s/file?id=wjVg/PLOSOne_formatting_sample_main_body.pdf and 

https://journals.plos.org/plosone/s/file?id=ba62/PLOSOne_formatting_sample_title_authors_affiliations.pdf

2. Please include captions for your Supporting Information files at the end of your manuscript, and update any in-text citations to match accordingly. Please see our Supporting Information guidelines for more information: http://journals.plos.org/plosone/s/supporting-information. 

3. If the reviewer comments include a recommendation to cite specific previously published works, please review and evaluate these publications to determine whether they are relevant and should be cited. There is no requirement to cite these works unless the editor has indicated otherwise. 

4. Please review your reference list to ensure that it is complete and correct. If you have cited papers that have been retracted, please include the rationale for doing so in the manuscript text, or remove these references and replace them with relevant current references. Any changes to the reference list should be mentioned in the rebuttal letter that accompanies your revised manuscript. If you need to cite a retracted article, indicate the article’s retracted status in the References list and also include a citation and full reference for the retraction notice.

[Note: HTML markup is below. Please do not edit.]

Reviewers' comments:

Reviewer's Responses to Questions

**Comments to the Author**

1. Is the manuscript technically sound, and do the data support the conclusions?

Reviewer #1: Yes

Reviewer #2: Yes

**********

2. Has the statistical analysis been performed appropriately and rigorously?

Reviewer #1: Yes

Reviewer #2: Yes

**********

3. Have the authors made all data underlying the findings in their manuscript fully available?

The PLOS Data policy

Reviewer #1: Yes

Reviewer #2: Yes

**********

4. Is the manuscript presented in an intelligible fashion and written in standard English?

Reviewer #1: Yes

Reviewer #2: Yes

**********

Reviewer #1: I congratulate the authors for this very interesting manuscript. However I have some comments:

1. Methodology: at the search strategy and studies eligibility criteria, the authors mention studies that report the prevalence and patterns of substance use in West Africa (lines 94,95 and 111, 112). Could the authors clarify the link with the current research topic which is mobile phone follow-up?

2. Discussion: I think that the suggestion to use WhatsApp (and similar platforms) needs to be better explained by the authors. The limitation of such platforms is the necessity for the user to have a smartphone and access to internet, which is variable in SSA, with limited access for people from low socioeconomic background and/or living in rural areas.

Reviewer #2: Overall this is an interesting article and a lot of effort was put into the analysis. It is however somewhat lacking in the discussion area and claims to share more insight than it does.

1. In the abstract and the text you write that:

“Retention rates were highest in Kenya (96%) and Nigeria (87%), while countries like Cameroon reported a loss rate of 42%.”

and

“Risk of bias assessments revealed that 81% of observational studies were low risk, while 69% of experimental studies were rated high quality.”

I would suggest both sentences be made into two sentences for clarity – otherwise the reader tries to compare retention and loss, or risk and high quality.

2. The Search strategy and Studies Eligibility Criteria both mention West Africa – is it not SSA?

3. You mention in the methods and results sections that text messages were sometimes included. It should be made clear in the abstract and introduction of the paper that text messages were included in some studies - text and phone calls use different software and sustain different human/tech costs.

4. Please also make clear if the phone calls were all via a health worker (human), an automated system or a combination of both.

5. Please define: Retention Rate, Acceptability, Feasibility, Follow-up Rates early in the paper

6. PICOS misspelled lines 114, 125, 129

7. Table 2: Can you please categorize the papers further? There is a column for ‘Study settings’. We need a column for type of health program e.g. chronic disease management, maternal health programs

8. You reference ‘mHealth infrastructure’ in lines 419 and 466. This is this something different than simply network connectivity’? Clarity needed.

9. Lines 425-426: you write ‘Conversely, infrequent follow-ups correlated with lower retention rates, emphasizing the dual importance of both frequency and quality of communication?’ There is nothing mentioned on quality – please clarify.

10. Line 431-433: you write “This review identified key barriers to implementing phone call follow-ups, including network connectivity issues, outdated or incorrect contact information, and participant relocations. These challenges, specific to resource-constrained environments, highlight the need for robust participant management strategies.” Are outdated and or incorrect contact information and participant relocations actually specific to resource-constrained environments? And can you give example(s) of a robust participant management strategy?

11. Line 435: you write “Additionally, integrating alternative communication platforms like WhatsApp could provide more accessible, cost effective options to improve follow-up success rates [70, 73-76].” This seems to be your big takeaway, so more needs to be added on why this is beneficial and how implementers could take this advice into account.

12. Line 463: You write “The study highlights actionable strategies to address barriers to retention, offering practical implications for policymakers and healthcare providers.” I do not think the discussion shares enough actionable strategies. More examples and context from the reviewed studies would strengthen this article.

**********

what does this mean? ). If published, this will include your full peer review and any attached files.

If you choose “no”, your identity will remain anonymous but your review may still be made public.

**Do you want your identity to be public for this peer review?** For information about this choice, including consent withdrawal, please see our Privacy Policy

Reviewer #1: No

Reviewer #2: No

**********

[NOTE: If reviewer comments were submitted as an attachment file, they will be attached to this email and accessible via the submission site. Please log into your account, locate the manuscript record, and check for the action link "View Attachments". If this link does not appear, there are no attachment files.]

While revising your submission, please upload your figure files to the Preflight Analysis and Conversion Engine (PACE) digital diagnostic tool, https://pacev2.apexcovantage.com/ . PACE helps ensure that figures meet PLOS requirements. To use PACE, you must first register as a user. Registration is free. Then, login and navigate to the UPLOAD tab, where you will find detailed instructions on how to use the tool. If you encounter any issues or have any questions when using PACE, please email PLOS at figures@plos.org

# Author comment: Author response to Decision Letter 1

**DOI:** 10.1371/journal.pone.0334894.r003

30 Sep 2025

Response to Reviewer 1

Reviewer #1: I congratulate the authors for this very interesting manuscript. However I have some comments:

1. Methodology: at the search strategy and studies eligibility criteria, the authors mention studies that report the prevalence and patterns of substance use in West Africa (lines 94,95 and 111, 112). Could the authors clarify the link with the current research topic which is mobile phone follow-up?

Response:

We thank the reviewer for pointing out this important clarification.

We acknowledge that the previous phrasing of our eligibility criteria may have unintentionally created ambiguity regarding the scope of our review. The initial mention of "prevalence and patterns of substance use" was residual from an earlier draft and was not aligned with the current focus of this review.

Accordingly, we have revised the eligibility criteria and search strategy sections (Lines XX–XX) to explicitly reflect that this systematic review is focused solely on studies that employed mobile phone follow-up strategies in health interventions in West Africa, regardless of the disease or health condition being addressed.

2. Discussion: I think that the suggestion to use WhatsApp (and similar platforms) needs to be better explained by the authors. The limitation of such platforms is the necessity for the user to have a smartphone and access to internet, which is variable in SSA, with limited access for people from low socioeconomic background and/or living in rural areas.

Response:

Thank you for this valuable observation. We agree that while platforms like WhatsApp offer low-cost communication options, their effectiveness is indeed constrained by the need for smartphones and reliable internet access—both of which remain unevenly distributed across Sub-Saharan Africa, particularly among rural and low-income populations.

We have revised the relevant section of the discussion to reflect this limitation and to emphasize that WhatsApp and similar platforms should be considered as complementary tools, rather than replacements, within a broader multi-modal follow-up strategy tailored to local context.

Revised Text in Manuscript:

“Additionally, integrating alternative communication platforms like WhatsApp could provide cost-effective options to improve follow-up success rates in urban and semi-urban areas. However, their utility may be limited in rural settings due to smartphone ownership and internet connectivity constraints. Future studies should assess the feasibility of blended approaches—combining basic mobile phone calls with app-based platforms—based on local infrastructure and population access.”

Response to Reviewer 2

Reviewer #2: Overall this is an interesting article and a lot of effort was put into the analysis. It is however somewhat lacking in the discussion area and claims to share more insight than it does.

1. In the abstract and the text you write that:

“Retention rates were highest in Kenya (96%) and Nigeria (87%), while countries like Cameroon reported a loss rate of 42%.”

and

“Risk of bias assessments revealed that 81% of observational studies were low risk, while 69% of experimental studies were rated high quality.”

I would suggest both sentences be made into two sentences for clarity – otherwise the reader tries to compare retention and loss, or risk and high quality.

Response:

Thank you for your insightful comment. We agree that separating the statements will improve clarity and prevent unintended comparisons. We have revised the relevant sentences in the abstract as follows:

“Retention rates were highest in Kenya (96%) and Nigeria (87%). In contrast, countries like Cameroon reported a high participant loss rate of 42%.”

“Risk of bias assessments showed that 81% of observational studies were rated as low risk. Additionally, 69% of experimental studies were assessed as high quality.”

2. The Search strategy and Studies Eligibility Criteria both mention West Africa – is it not SSA?

Response:

Thank you for pointing this out. We have reviewed and corrected all instances to reflect Sub-Saharan Africa (SSA) in both the Search Strategy and Studies Eligibility Criteria sections to ensure consistency with the scope of the review.

3. You mention in the methods and results sections that text messages were sometimes included. It should be made clear in the abstract and introduction of the paper that text messages were included in some studies - text and phone calls use different software and incur different human/tech costs.

Response:

Thank you for this valuable suggestion. We agree that phone calls and text messages involve different implementation considerations and costs. While our review primarily focused on studies using phone call follow-up interventions, a small number of the included studies incorporated text messaging alongside phone calls.

To improve clarity and transparency, we have now updated the Introduction to briefly note that some studies included text messaging as part of a broader phone-based intervention. We have also added a line in the Abstract to acknowledge this, while maintaining the focus on phone call follow-up as the primary intervention of interest.

4. Please also make clear if the phone calls were all via a health worker (human), an automated system or a combination of both.

Response:

Thank you for this important observation. We have clarified in both the Methods and Results sections that the mode of phone call delivery (i.e., human vs. automated) was not consistently reported across the included studies. As such, we did not provide a separate analysis or summary on this aspect.

5. Please define: Retention Rate, Acceptability, Feasibility, Follow-up Rates early in the paper

Response:

Thank you for this important suggestion. We have now provided clear operational definitions of the key outcomes — retention rate, acceptability, feasibility, and follow-up rate — in the Methods section under a new subsection titled “Study Outcomes.” This placement ensures that readers are aware of how these outcomes were conceptualized and measured before encountering the results.

6. PICOS misspelled lines 114, 125, 129

Response:

Thank you for pointing this out. We have corrected the spelling of PICOS in all indicated lines. Regarding the “Comparator” component, we have updated the PICOS framework table and narrative to reflect that no comparator was applicable in our review, and this is now clearly stated as “Comparator: Not applicable.”

7. Table 2: Can you please categorize the papers further? There is a column for ‘Study settings’. We need a column for type of health program e.g. chronic disease management, maternal health programs

Response

Thank you. We have added a column for health programs.

8. You reference ‘mHealth infrastructure’ in lines 419 and 466. This is this something different than simply network connectivity’? Clarity needed.

Response:

Thank you for your helpful comment. We agree that the term “mHealth infrastructure” may have introduced ambiguity, especially since the primary focus of our review is on phone call follow-up interventions. To address this, we have revised the text to more accurately reflect our intended meaning.

9. Lines 425-426: you write ‘Conversely, infrequent follow-ups correlated with lower retention rates, emphasizing the dual importance of both frequency and quality of communication?’ There is nothing mentioned on quality – please clarify.

Response

Thank you for your thoughtful comment. We acknowledge that the term “quality of communication” could be clearer in the context of our study. In this review, we used it to refer to the interpersonal effectiveness of the follow-up interaction, such as the clarity, empathy, and responsiveness of the health worker during the phone call, rather than technical aspects like call quality or signal strength.

To address this, we have revised the text to clarify that “quality of communication” refers to how well the phone interaction fosters trust, understanding, and patient engagement, which is particularly important in contexts where patients may have concerns or questions that require human reassurance. We have made this distinction explicit in the relevant paragraph to improve clarity and alignment with the study’s focus on human-led phone call follow-ups.

10. Line 431-433: you write “This review identified key barriers to implementing phone call follow-ups, including network connectivity issues, outdated or incorrect contact information, and participant relocations. These challenges, specific to resource-constrained environments, highlight the need for robust participant management strategies.” Are outdated and or incorrect contact information and participant relocations actually specific to resource-constrained environments? And can you give example(s) of a robust participant management strategy?

Response:

Thank you for this insightful comment. We agree that outdated or incorrect contact information and participant relocations are not unique to resource-constrained settings and may occur in various contexts. However, in resource-limited environments, these challenges are often compounded by systemic factors such as inconsistent record-keeping practices, lack of electronic medical records, and limited mobile phone ownership or SIM card stability (e.g., frequent number changes). We have revised the sentence to reflect this nuance more accurately.

11. Line 435: you write “Additionally, integrating alternative communication platforms like WhatsApp could provide more accessible, cost effective options to improve follow-up success rates [70, 73-76].” This seems to be your big takeaway, so more needs to be added on why this is beneficial and how implementers could take this advice into account.

Response:

Thank you for this helpful observation. We agree that the mention of alternative communication platforms like WhatsApp warrants further elaboration, especially given its widespread use and growing utility in healthcare delivery across Sub-Saharan Africa.

We have revised the text to better explain why integrating platforms such as WhatsApp may be beneficial. Specifically, we note that WhatsApp allows for asynchronous communication, reduced costs compared to voice calls, and the ability to share multimedia content (e.g., images, reminders, or educational material), which can enhance patient engagement. Furthermore, WhatsApp is already familiar to both healthcare providers and patients in many African settings, making it a feasible and acceptable tool for follow-up.

12. Line 463: You write “The study highlights actionable strategies to address barriers to retention, offering practical implications for policymakers and healthcare providers.” I do not think the discussion shares enough actionable strategies. More examples and context from the reviewed studies would strengthen this article.

Response:

Thank you for the helpful suggestion. We have revised the discussion to include concrete examples of actionable strategies such as verifying contacts, scheduled follow-ups, small incentives, local language use, WhatsApp integration, and dedicated follow-up staff, to enhance practical relevance for implementers.

Attachment Submitted filename: Response to Reviewer 2.pdf

# Editor report: Decision Letter 1

**DOI:** 10.1371/journal.pone.0334894.r004

**Authors:** Francis Xavier Kasujja

6 Oct 2025

Evaluating Phone Call Follow-Ups in Sub-Saharan Africa:  A Systematic Review and Meta-Analysis

PONE-D-25-04857R1

Dear Dr. Akinsolu,

We’re pleased to inform you that your manuscript has been judged scientifically suitable for publication and will be formally accepted for publication once it meets all outstanding technical requirements.

Within one week, you’ll receive an e-mail detailing the required amendments. When these have been addressed, you’ll receive a formal acceptance letter and your manuscript will be scheduled for publication.

An invoice will be generated when your article is formally accepted. Please note, if your institution has a publishing partnership with PLOS and your article meets the relevant criteria, all or part of your publication costs will be covered. Please make sure your user information is up-to-date by logging into Editorial Manager at Editorial Manager®  and clicking the ‘Update My Information' link at the top of the page. For questions related to billing, please contact billing support .

If your institution or institutions have a press office, please notify them about your upcoming paper to help maximize its impact. If they’ll be preparing press materials, please inform our press team as soon as possible -- no later than 48 hours after receiving the formal acceptance. Your manuscript will remain under strict press embargo until 2 pm Eastern Time on the date of publication. For more information, please contact onepress@plos.org.

Kind regards,

Francis Xavier Kasujja

Academic Editor

PLOS ONE

# Editor report: Acceptance letter

**DOI:** 10.1371/journal.pone.0334894.r005

**Authors:** Francis Xavier Kasujja

PONE-D-25-04857R1

PLOS ONE

Dear Dr. Akinsolu,

I'm pleased to inform you that your manuscript has been deemed suitable for publication in PLOS ONE. Congratulations! Your manuscript is now being handed over to our production team.

At this stage, our production department will prepare your paper for publication. This includes ensuring the following:

* All references, tables, and figures are properly cited

* All relevant supporting information is included in the manuscript submission,

* There are no issues that prevent the paper from being properly typeset

You will receive further instructions from the production team, including instructions on how to review your proof when it is ready. Please keep in mind that we are working through a large volume of accepted articles, so please give us a few days to review your paper and let you know the next and final steps.

Lastly, if your institution or institutions have a press office, please let them know about your upcoming paper now to help maximize its impact. If they'll be preparing press materials, please inform our press team within the next 48 hours. Your manuscript will remain under strict press embargo until 2 pm Eastern Time on the date of publication. For more information, please contact onepress@plos.org.

You will receive an invoice from PLOS for your publication fee after your manuscript has reached the completed accept phase. If you receive an email requesting payment before acceptance or for any other service, this may be a phishing scheme. Learn how to identify phishing emails and protect your accounts at https://explore.plos.org/phishing.

If we can help with anything else, please email us at customercare@plos.org.

Thank you for submitting your work to PLOS ONE and supporting open access.

Kind regards,

PLOS ONE Editorial Office Staff

on behalf of

Dr. Francis Xavier Kasujja

Academic Editor

PLOS ONE
